# Brain functional alteration and cognitive performance in cardiovascular diseases: a systematic review of fMRI studies

**DOI:** 10.3389/fneur.2024.1425399

**Published:** 2024-10-15

**Authors:** Syeda Humayra, Noorazrul Yahya, Chai Jia Ning, Imtiyaz Ali Mir, Abdul Latiff Mohamed, Hanani Abdul Manan

**Affiliations:** ^1^Makmal Pemprosesan Imej Kefungsian (Functional Image Processing Laboratory), Department of Radiology, University Kebangsaan Malaysia, Kuala Lumpur, Malaysia; ^2^Diagnostic Imaging and Radiotherapy Program, Faculty of Health Sciences, School of Diagnostic and Applied Health Sciences, Universiti Kebangsaan Malaysia, Kuala Lumpur, Malaysia; ^3^Department of Radiology and Intervention, Hospital Pakar Kanak-Kanak (UKM Specialist Children’s Hospital), Universiti Kebangsaan Malaysia, Kuala Lumpur, Malaysia; ^4^Department of Physiotherapy, M Kandiah Faculty of Medicine and Health Sciences, Universiti Tunku Abdul Rahman, Kajang, Malaysia; ^5^Faculty of Health Sciences, Lincoln University College, Petaling Jaya, Malaysia; ^6^Faculty of Medicine, University of Cyberjaya, Cyberjaya, Malaysia

**Keywords:** brain functional alteration, cognition, CVDs, fMRI, task-based, resting-state

## Abstract

**Background:**

Functional magnetic resonance imaging (fMRI) is a useful tool to evaluate brain inefficiencies secondary to cardiovascular diseases (CVDs); nevertheless, limited fMRI studies have been conducted to investigate the effect of CVDs on brain functional changes and cognitive function. This systematic review aims to explore, synthesise, and report fMRI outcomes (resting state and task-based) and cognitive performance in patients with CVDs.

**Methods:**

Two reviewers independently searched published literature until April 2024 on ScienceDirect, PubMed, Web of Science, and ClinicalTrials.gov adhering to the PRISMA protocol. A total of 26 eligible studies were considered for full-text screening, of which 10 were included in this review. The methodological quality was assessed by mixed methods appraisal tool and was reported as empirically fair.

**Results:**

Among 336 subjects with CVDs, aged between 49.90 ± 6.10 to 72.20 ± 5.70 years, the majority had coronary artery diseases (n = 177, 52.68%) and hypertension (n = 200, 59.52%), and approximately half of them were females (n = 169, 50.30%). Based on the qualitative synthesis, subjects with CVDs demonstrated an increased cognitive decline (reduced Mini-Mental State Examination/Montreal Cognitive Assessment mean values) and attenuated task performance (lower mean 2-back task scores and slower reaction time). Results also indicated impaired brain activity at the supplementary motor area associated with poor ejection fraction; reduced default mode network suppression linked to high low-density lipoprotein cholesterol; lower regional homogeneity and amplitude of low-frequency fluctuation values; and reduced functional connectivity. In summary, alterations in brain networks connectivity may have contributed to an impaired cognitive performance in patients with cardiovascular diseases.

**Conclusion:**

It can be extrapolated that CVDs tend to alter the brain network connectivity and result in cognitive impairment and poorer task performance. However, for future imaging studies, more stringent and homogenous demographic data are highly recommended.

## Introduction

Cardiovascular disease (CVDs) are an umbrella term that includes numerous heart and blood vessel disorders, such as coronary artery diseases (CADs), myocardial infarction (MI), heart failure (HF), and hypertension (HTN) (1). Over the last three decades, CVDs have been the world’s largest contributor to mortality and disability, with an increased prevalence from 271 million in 1990 to 523 million in 2019, making it a significant public health concern (2). An increasing body of research indicates a direct connection between the heart and brain functioning. Multiple brain disorders, including stroke, dementia, cerebral small vessel disease, and cognitive impairment (CI), tend to show a pathophysiological association with CVDs (3, 4). CI is a clear deterioration of functioning in one or more of the key cognitive domains including perceptual-motor function, language, executive function, learning and memory, complex attention, and social cognition. The prevalence of CVDs and CI is remarkably higher in advancing age, which leads to additional healthcare burden and diminished quality of life (QoL) (5).

In clinical practice, coronary heart diseases (CHDs) are one of the most common CVDs. Data has shown a significant link between CHDs and structural and functional alterations in the central nervous system (CNS) (6). In fact, clinical evidence indicates a strong connection between cardiac malfunction and cerebral injury (7). Heart failure (HF), which is a rapidly growing phenomenon in the Western world, is most likely attributable to the existence of CHDs (8). The severity of HF and brain involvement influences long-term adverse outcomes, in addition to impaired brain function and changes in cardiac hemodynamics, regardless of the comorbidities and cardiovascular risk factors (CVRFs) (8). The clinical diagnostic and evaluation criteria, such as the Framingham Risk Score (FRS), mostly concentrate on demographics, health behaviours, and peripheral physiological indicators to predict the risk of CVDs, with no particular emphasis on the brain-related biomarkers (9). However, mounting evidence suggests that the brain’s anatomical and functional characteristics are related to the risk factors of CVDs (e.g., high blood pressure and cholesterol levels), and may serve as precursors to dementia, brain aging, and neurocognitive decline (9). Furthermore, the risk factors for cardiovascular and cerebrovascular illnesses have been often linked to pathological brain alterations resulting in CI, regardless of the presence or absence of clinically significant vascular events (10).

According to human neuroimaging findings, the brain’s structure and function may have a serious impact on the progression of CVDs (9). The use of advanced imaging technologies in today’s medical practices is expanding tremendously. Researchers have discovered certain brain-behaviour linkages and predicted a number of self-management behaviours using functional magnetic resonance imaging (fMRI), a method used to assess neural activity (11). Functional brain changes have been previously reported using fMRI, whereof, resting-state functional magnetic resonance imaging (rs-fMRI) study (12) revealed a weakened intra-system functional connectivity (FC), and task-based functional magnetic resonance imaging (tb-fMRI) evaluated the working memory (WM) performance in older healthy adults using a verbal N-back task (13). Healthy cognitive aging (HCA) has distinct patterns of neural brain activation (14) and WM function in mild CI (15), in comparison to individuals with CVDs (16, 17).

Interventions to optimise cardiac function might have implications for the preservation of brain function, therefore, clinicians should also focus on the cognitive performance and brain condition of patients with CVDs, which is consistent with the American Heart Association’s current guidelines since CVRFs contribute to evident vascular CI. Cognitive impairments including attention and learning deficits, memory loss, and, to a lesser extent, language impairment and decreased visual–spatial ability are some common cognitive changes reported in this target population. Additionally, the cognitive function in patients with CVDs is much lower than healthy controls (HCs), and persists even after adjustment of age (8).

Although CVDs have been associated with cognitive deficits in the absence of clinically identified stroke, and fMRI has been a useful tool to further characterise the brain inefficiencies secondary to CVDs (18); nevertheless, limited fMRI studies have been conducted to investigate the effect of CVDs on brain functional changes and cognition. Therefore, this systematic review aims to explore the current body of literature and synthesise adequate information regarding the fMRI outcomes (rs-fMRI and tb-fMRI) and cognitive alterations in patients with CVDs.

## Methods

### Search strategy

This systematic review followed the Preferred Reporting Items for Systematic Reviews and Meta-Analyses 2020 (PRISMA 2020) and past studies (19–23) as the reporting guidelines. The review protocol has been registered in PROSPERO (CRD42023456259). Two reviewers systematically searched all relevant articles published until April 2024 on four databases (PubMed, Web of Science, ScienceDirect, and ClinicalTrials.gov). The fMRI studies relevant to CVDs and cognitive function were screened and selected. CVDs were searched along with their subgroups such as CADs, HF, CHDs, ischaemic heart diseases (IHDs), and/or the cardiovascular risk factors. The key search terms included “functional magnetic resonance imaging,” “fMRI,” “cardiovascular diseases,” “CVDs,” and “cognition,” which were connected by Boolean operators (OR/AND). The detailed search strategies and keywords (Supplementary Table S1), study selection criteria for inclusion and exclusion (Supplementary Tables S2, S3), can be found in Supplementary material. The search strategy was filtered to identify original research studies in English language conducted on human subjects. The bibliography of all included studies was further reviewed to identify any additional studies missed during the initial search, and this was also performed independently by two reviewers.

### Inclusion and exclusion criteria

All fMRI studies (both resting state and task-based) related to CVDs, their subgroups, or related risk factors such as hypertension (HTN), type II diabetes mellitus (T2DM), dyslipidaemia (DLD), hypercholesterolemia (HCL), and smoking etc., were considered for inclusion. Only original research studies on adults (≥18 years old) with confirmed CVDs status was included. The PICOS framework for study inclusion has been summarised (Table 1). On the contrary, individuals with chronic comorbid status including liver/ lung/ kidney diseases, and neurological, musculoskeletal, and psychological conditions (e.g., dementia, anxiety, and depression), cancer, and other inflammatory diseases were excluded since these patient groups may be susceptible to pre-existing cognitive impairments. In addition, individuals with pre-existing cerebrovascular diseases and congenital heart diseases were excluded since they are prone to developing structural changes in the brain and heart that may interfere with the study outcomes. The exclusion criteria further included preprints, conference abstracts, systematic reviews and meta-analyses, and brief reports. Studies reporting structural MRI, or other imaging modalities rather than fMRI, and conducted among children/infants, pregnant women, or in individuals with an underlying history of traumatic brain injury, substance or alcohol use, and MRI contraindications were also excluded.

**Table 1:** PICOS framework.

(P)	Population	Adults with history of CVDs such as CADs, HF, CHDs, IHDs, and/or CVDs risk factors (atherosclerosis, hypertension, obesity, dyslipidaemia, T2DM, smoking)
(I)	Intervention	Functional magnetic resonance imaging (resting state fMRI and task-based fMRI)
(C)	Comparison	Healthy adult control
(O)	Outcome	Brain functional changes and neuropsychological assessment (RSN/ brain activity/ connectivity, cognition, working memory, attention and reaction time)
(S)	Study design	Original research articles (RCT, prospective, retrospective, and cross-sectional study)

CADs, coronary artery diseases; CHDs, coronary heart diseases; CVDs, cardiovascular diseases; fMRI, functional magnetic resonance imaging; HF, heart failure; IHDs, ischaemic heart diseases; RCT-randomized controlled trial; RSN, resting state network; T2DM, type II diabetes mellitus.

### Study selection

All searched articles were imported into EndNote for repeated inspection. After deleting duplicate studies, two independent reviewers screened the titles and abstracts of all the articles and selected potentially relevant studies. Then both the co-authors separately reviewed the full-text studies and, according to the inclusion/exclusion criteria, decided on the relevant studies to be included in the review. Any disagreement between the two authors was resolved through mutual consensus of a third co-author/reviewer.

### Data extraction

Studies meeting the inclusion criteria were processed for data extraction. Two reviewers independently extracted and recorded data in a consistent way using a standardized data extraction form on an excel spreadsheet. The authors used a predefined data collection form (Table 2) to extract required information, including first author and publication year, location/region, study design, sample size, age range, CVDs entity, the type of fMRI, neuropsychological assessment, and brain regions of interest. The primary outcome of interest were the brain functional changes and neuropsychological assessment in patients with CVDs. The secondary outcomes were the demographic and clinical characteristics (CVDs vs. HCs). The fMRI outcomes for rs-fMRI and tb-fMRI included resting state network (RSN)/ brain activity/ connectivity, cognition, working memory, attention and reaction time.

**Table 2:** Summary characteristics of included studies.

Study	Location	Study design	Sample size	Age range (years)	CVDs entity	Comorbidity	fMRI	Neuropsychological assessment (Mean ± SD)	Brain regions of interest (ROIs)
Qin et al. (31)	Jinan, China	Cross-sectional	48	NR	CAD	3	Resting state	MoCA (21.86 ± 4.21/26.80 ± 1.90) MMSE (24.81 ± 2.56/ 27.29 ± 1.98)	Superior frontal gyrus. Parahippocampal cortex, and medial temporal gyrus
Wei et al. (7)	Nanjing, China	Cross-sectional	42	47–69	CHD	1	Resting state	MoCA (>26)	Bilateral thalami and left hemisphere
Sun et al. (17)	Yantai, China	Prospective	144	52–70	CHD	2	Resting state	MoCA (22.15 ± 2.41/ 23.94 ± 1.71) MMSE (23.78 ± 2.01/ 26.82 ± 2.01)	Medial superior frontal gyrus (SFGmed) and left middle temporal gyrus (TPOmid)
Zhang et al. (29)	Hangzhou, China	Cross-sectional study	45	35–80	CAD	3	Resting state	MoCA (17.18 ± 3.13/ 28.18 ± 1.33) MMSE (20.45 ± 4.01/26.73 ± 1.74) Visual acuity (0.13 ± 0.10/−0.14 ± 0.35)	Right precuneus gyrus, left supramarginal gyrus, left angular gyrus, and left middle cingulum gyrus
Lin et al. (25)	Xiamen, China	Cohort	60	40–70	CHD	2	Resting state	NR	Left precentral/ postcentral gyrus, and right inferior cerebellum
Meusel et al. (32)	Toronto, Canada	Cross-sectional study	30	65–85	High risk of CVDs (FRS >20) CVD risk factors (HTN, T2DM, DLD)	3	Task-based	WASI (61.20 ± 8.50) Shipley vocabulary (58.80 ± 6.50)	Medial and lateral parietal and temporal regions, and right anterior cingulate and medial frontal gyrus
Chuang et al. (26)	Baltimore, Maryland, USA	Cross-sectional study	60	60–74	High risk of CVDs (FRS =19.7) CVDs risk factors (HTN, T2DM, and smoking)	2	Task-based	MMSE (28.50 ± 1.40) TMT part A (43.4 ± 14.2) TMT part B (120.3 ± 66.3)	Left middle temporal lobe, and left inferior parietal lobe (supramarginal gyrus)
Haley et al. (16)	Austin, Texas, USA	Cross-sectional	49	40–60	CVDs risk factors (HTN, DLD, obesity, T2DM, and smoking)	>3	Task-based	CDS (114.46 ± 10.5) BDI-II (score NR) MMSE (28.40 ± 1.40) WASI (114.4 ± 10.5) WASI vocabulary (64.4 ± 9.1) Category fluency for animals (24.6 ± 5.1) TMT A (28.4 ± 8.4) TMT B (68.0 ± 25.9) COWAT (38.3 ± 11.0) Pegs-D (75.9 ± 14.8) WAIS-III (16.7 ± 3.7) Visual–spatial memory (CVLT-II, RCF)	Left middle frontal gyrus, left medial frontal gyrus, right superior parietal lobule, left inferior parietal lobule, left middle frontal gyrus, right superior frontal gyrus, right middle frontal gyrus, right inferior frontal gyrus.
Irani et al. (18)	Philadelphia, Pennsylvania, USA	Prospective study	17	57–84	Angina pectoris = 4, HF = 4, arrhythmia = 4, cardiac surgery = 5, CAD = 6, history of MI = 8, and HTN = 10	1	Task-based	MMSE (29.00 ± 2.00) DRS (140 ± 3)	Dorsolateral prefrontal, posterior parietal, supplementary motor area, medial prefrontal gyrus, insula, and posterior cingulate gyrus
Haley et al. (30)	Rhode Island, USA	Longitudinal study	12	55–85	T2DM = 1, valve surgery = 2, HF = 2, CAD = 3, smoking = 4, MI = 5, HTN = 7, and HCL = 7	>3	Task-based	MMSE (29.00 ± 0.95) DRS score (139.75 ± 2.86) BDI score (3.73 ± 2.57)	Left middle frontal gyrus, left medial frontal gyrus, right superior parietal lobule, left inferior parietal lobule, left middle frontal gyrus, right superior frontal gyrus, right middle frontal gyrus, right inferior frontal gyrus.

BDI, Beck Depression Inventory; CAD, coronary artery disease; CDS, cognitive difficulties scale; CHD, coronary heart disease; COWAT, Controlled Oral Word Association Test; CVDs, cardiovascular diseases; CVLT, California Verbal Learning Test; DLD, dyslipidemia; DRS, Dementia Rating Scale; fMRI, functional magnetic resonance imaging; HCL, hypercholesterolemia; HF, heart failure; MI, myocardial infarction; MMSE, Mini Mental Status Exam; MoCA, Montreal Cognitive Assessment; NR, not reported; Pegs-D, Grooved Pegboard; Dominant Hand; RCF, Rey Complex Figure Test; TMT, Trail Making Test; T2DM, type 2 diabetes mellitus; WAIS, Wechsler Adult Intelligence Scale; WASI, Wechsler Abbreviated Scale of Intelligence.

### Methodological assessment criteria

The methodological quality of each study was independently assessed by two reviewers using the Mixed Methods Appraisal Tool (MMAT) (24). The MMAT is a critical appraisal tool that is designed for the appraisal stage of systematic mixed studies reviews. It permits appraisal of the methodological quality of five categories of studies: qualitative research, randomized controlled trials, non-randomized studies, quantitative descriptive studies, and mixed methods studies. The methodological criteria of the included studies were assessed through quantitative non-randomized studies’ (5-items) for cohorts and cross-sectional studies. The screening questions for both types of study assessment included only three responses: “Yes”, “No”, and “Cannot tell”. Responding “No” or “Cannot tell” to more than one question on the checklist indicates that the paper is not an empirical study, and thus cannot be appraised using the MMAT. The “Cannot tell” response category means that the paper do not report appropriate information to answer “Yes” or “No”, or that report unclear information related to the criterion. Rating “Cannot tell” could lead to look for companion papers, or contact authors to ask more information or clarification when needed.

## Results

### Search results

**Figure 1:**
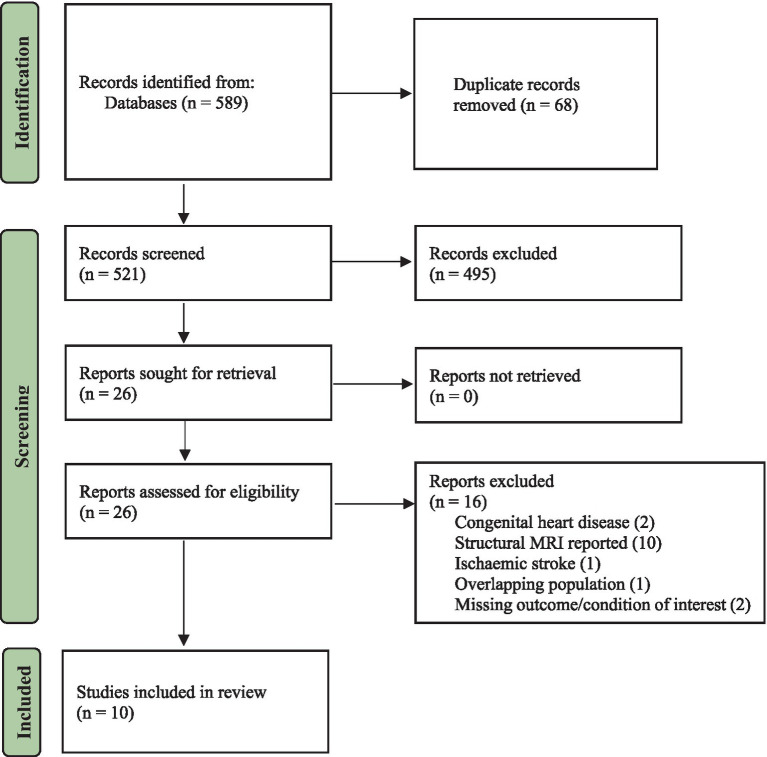
PRISMA flowchart.

A total of 589 articles were found in the databases after running the systematic search. In addition, a manual search of the study references yielded two more relevant articles. After the removal of duplicates, the titles and abstracts of 521 articles were screened and read, and 495 irrelevant studies were excluded. A total of 26 eligible studies were considered for full-text screening, of which 16 were excluded. The study screening process and inclusion or exclusion of studies can be found in the PRISMA flow chart (see Figure 1). In summary, 10 articles involving 507 subjects (CVDs = 336, control = 171) between the age of 35–85 years were included in the final qualitative synthesis.

### Study characteristics

The main characteristics of the fMRI studies conducted among CVDs population are summarized in Table 2. Among the included studies (n = 10) published between 2007 to 2023, most of them presented with CHDs or CADs (n = 5) as the major CVDs entity. Most of the studies reported at least 3 or more comorbidities (n = 5). Whereas, in terms of CVDs risk factors, HTN was reported across all studies (n = 10), followed by T2DM (n = 8), smoking (n = 6), and dyslipidaemia (n = 5). Out of 10 fMRI studies, 5 investigated the task-based outcomes, and others explored the resting state (n = 5). The majority of these studies were conducted in USA (n = 4) and China (n = 5), and only one in Canada. Most of them adopted a cross-sectional design (n = 6), followed by prospective (n = 2), longitudinal (n = 1), and cohort (n = 1).

### Demographic and clinical characteristics

Among the 336 CVDs subjects, the proportion of female (n = 169) versus male patients (n = 167) was almost similar, and had no significant difference. The mean age of subjects ranged between 49.90 ± 6.10 to 72.20 ± 5.70 years. The minimum vs. maximum educational years were 5.09 ± 1.41 vs. 15.60 ± 2.90, respectively. Based on the information reported in all ten studies, a total of 200 subjects with hypertension (n = 10), 86 with diabetes (n = 8), 71 with dyslipidaemia (n = 5), and 54 smokers (n = 6) were recorded. CVDs subjects showed comparatively diminished cognitive scores than HCs in both MoCA (17.18 ± 3.13, 28.18 ± 1.33) and MMSE (20.45 ± 4.01, 26.73 ± 1.74). In terms of clinical characteristics, 5 studies reported BMI, whereof, the BMI in patients with CVDs ranged from 23.71 ± 2.62 (25) to 31.3 ± 6.4 (26). In addition, only 3 studies reported the mean LDL and HDL cholesterol values, and 3 studies included the mean ejection fraction (EF) percentile. In case of CVDs at-risk population studies, the Framingham risk score (FRS >20; FRS = 19.7) was provided in 2 studies out of 3. The summary of demographic and clinical characteristics is presented (Table 3).

**Table 3:** Demographic and clinical characteristics of patients with CVDs and healthy controls.

	Patients / healthy control													
Study	Sample size	Age in years (Mean ± SD)	Male (n)	Female (n)	Education (years)	Smoker (n)	Comorbid status (n)				Clinical characteristics (Mean ± SD)			
							HTN	T2DM	DLD	Obes/ HCL	BMI (kg/cm^2^)	LDL (mmol/L)	HDL (mmol/L)	EF (%)
Qin et al. (31)	24/24	58.8 ± 9.64/56.9 ± 12.2	12/15	12/9	12.6 ± 3.63 / 12.5 ± 3.85	10/13	12/16	11/6	15/11	NR	NR	NR	NR	NR
Wei et al. (7)	26/16	60.46 ± 8.85/57.25 ± 10.48	13/5	13/11	NR	NR	16/7	NR	NR	NR	24.25 ± 3.01/24.94 ± 3.93	2.62 ± 1.03/2.29 ± 0.91	NR	65.38 ± 4.06/ 65.38 ± 7.07
Sun et al. (17)	71/73	61.49 ± 8.32/ 60.68 ± 8.33	38/39	33/34	10.33 ± 2.42/ 11.12 ± 3.37	NR	41/30	13/8	NR	NR	26.63 ± 3.05/ 25.47 ± 3.40	3.25 ± 0.68/ 3.04 ± 0.54	1.21 ± 0.30/ 1.33 ± 0.31	>60
Zhang et al. (29)	22/23	59.91 ± 6.82/60.64 ± 6.79	17/16	5/7	5.09 ± 1.41/ 5.11 ± 1.01	11/14	7/11	20/22	18/17	NR	NR	NR	NR	>50
Lin et al. (25)	25/35	57.44 ± 7.18/53.74 ± 7.66	14/21	11/14	9.32 ± 4.77/ 10.03 ± 3.51	7/11	14/19	4/4	NR	NR	23.71 ± 2.62/23.66 ± 2.70	NR	NR	65.96 ± 5.93/65.46 ± 6.80
Meusel et al. (32)	30	72.20 ± 5.70	14	16	15.60 ± 2.90	NR	30	13	16	NR	26.10 ± 2.50	2.40 ± 1.00	1.60 ± 0.40	NR
Chuang et al. (26)	60	64.60 ± 3.70	15	45	13.90 ± 2.30	11	43	13	NR	NR	31.3 ± 6.4	NR	58.1 ± 16.9	NR
Haley et al. (16)	49	49.90 ± 6.10	25	24	15.1 ± 2.2	11	20	11	15	22	NR	NR	NR	NR
Irani et al. (18)	17	68.00 ± 8.00	11	6	15.0 ± 2.0	NR	10	NR	NR	NR	NR	NR	NR	60.00 ± 612.0
Haley et al. (30)	12	68.75 ± 6.89	8	4	15.17 ± 1.95	4	7	1	7	7	NR	NR	NR	NR

BMI, body mass index; DLD, dyslipidemia; EF, ejection fraction; HCL, hypercholesterolemia; HDL, high-density lipoprotein; HTN, hypertension; LDL, low-density lipoprotein; NR, not reported; Obes., obesity; T2DM, type II diabetes mellitus.

### Quality assessment of included studies

The methodological quality of studies was critically appraised using MMAT (24), which is a valid and reliable appraisal tool for systematic reviews (27, 28). All 10 studies passed the two screening questions and were then reviewed further. Clear descriptions of the target population with detailed justification of the inclusion and exclusion criteria were provided in all studies (n = 10). In addition, the variables were clearly defined and measured; the fMRI intervention outcomes and measurements were clearly justified and appropriate for answering the research question. However, in terms of reporting of outcomes, one study did not report the neuropsychological assessment tool and/or score, and failed to mention any absolute or standard cut-off value for acceptable complete outcome data. Whereas the other only reported a cut-off value of MoCA without any clear indication of mean scores to differentiate the cognitive outcomes between CVDs and control group subjects. Although most of the studies (n = 5) tried to limit the confounding bias through appropriate methods such as matching and standardization, but others (n = 5) failed to control for confounders as the demographic (gender, education, ethnicity) and clinical characteristics (BMI, LDL, HDL, SBP, DBP, blood glucose, HBA1c, medication history, CVDs duration or diagnosis period, comorbidity, and smoking status) of study subjects were not standardized appropriately. During the study period, the participants were treated in a standardized manner that was appropriate for fMRI investigation. However, it was not possible to deduce whether the influence of confounders on the outcome of interest, were accounted for, or whether any unplanned co-exposures were present in control groups (n = 2). Nevertheless, the overall quality of the included studies was empirically fair when assessed by the MMAT because majority of the studies fulfilled the methodological criteria (Supplementary Table S4).

### Neuropsychological assessment

MMSE (n = 7) and MoCA (n = 4) were the most commonly practiced neuropsychological assessment tools across all studies either independently or collectively alongside other cognitive tests. The highest mean score attained in MMSE was 29.00 ± 2.00 (18) and the lowest score was 20.45 ± 4.01 (29). While in case of MoCA, the lowest score reported was 17.18 ± 3.13 and the highest was 28.18 ± 1.33 (29). Furthermore, it was noted that HCs demonstrated better cognitive functioning than individuals with CVDs or at risk of CVDs (17, 29). One study (16) specifically grouped neuropsychological measures into five cognitive domains and measured the test scores for each domain which are global cognitive functioning (MMSE and WASI Full Scale IQ), language functions (WASI Vocabulary Subtest and Category Fluency for Animals), visual–spatial abilities (RCF copy and WASI Matrix Reasoning Subtest), memory functions (CVLT-II immediate recall, delayed recall, and recognition discrimination, RCF immediate recall, delayed recall, and recognition discrimination), and finally attention-executive-psychomotor functions (Trail Making A and B time to completion, COWAT, WAIS-III Digit Span Subtest, and Grooved Pegboard-Dominant Hand time to completion). In addition, 2 studies (18, 30) also utilised the Dementia Rating Scale (DRS) in patients with CVDs and reported a score of 140 ± 3 and 139.75 ± 2.86, respectively. Whereas, the Beck Depression Inventory (BDI) was reported in 2 studies (16, 30) and the mean score (3.73 ± 2.57) was mentioned only in one (30).

## Functional MRI outcomes

### Resting state fMRI

Among the 10 studies, 5 of them performed resting-state fMRI, whereof, 2 studies (7, 25) reported sensorimotor networks (SMN), and another study (17) reported the default mode network (DMN). Wei et al. (7) investigated the thalamus-based functional connectivity (FC) patterns in patients with CHDs. FC analysis of the left thalamus revealed that CHDs subjects exhibited decreased FC patterns with the left supplementary motor area (SMA) and superior frontal gyrus (SFG) in comparison to HCs. Whereas, for the right thalamus, the researchers found reduced FC patterns with the left hemisphere, encompassing SMA, superior parietal gyrus (SPG), inferior parietal gyrus (IPG), middle cingulate cortex (MCC), lingual gyrus, and calcarine sulcus. In short, the study demonstrated a significant correlation between decreased functional lateralization of thalamic FC patterns and several subnetworks in the left hemisphere in patients with CHDs. Another study (31) found that patients with CADs had an increase in negative connectivity from parahippocampal cortex (PHC) and medial temporal gyrus (MTG) to SFG, along with a decrease in the strength of positive connectivity between PHC and MTG, and showed lower cognitive function compared to HCs. It was formulated that alterations in the connectivity of the brain networks may contribute to the cognitive impairment in CADs. Sun et al. (17) found decreased regional homogeneity (ReHo) values in bilateral medial superior frontal gyrus (SFGmed) and left middle temporal gyrus (TPOmid) in subjects with CHDs, and increased ReHo values in the right rolandic operculum (ROL) and right insula (INS). Decreased spontaneous brain activity in SFGmed correlated with CI in CHDs; whereas high coronary artery calcium (CAC) scores correlated with poor psychological measures and cognitive dysfunction. Three studies (17, 25, 29) utilised the amplitude of low-frequency fluctuation (ALFF) to examine the local spontaneous patterns during the resting state. Brain regions showed notable ALFF differences in patients with CADs, in which reduced ALFF values correlated with lower MoCA scores and reduced visual acuity (29). Sun et al. (17) found reduced fALFF values in the left and right SFGmed, left MTG and right superior temporal gyrus (TPOsup) in CHDs, and increased fALFF values in the left inferior occipital gyrus (IOG). While Lin et al. (25) found reduced fALFF values in the left precentral/postcentral gyrus, and increased fALFF values in the right inferior cerebellum. In summary, subjects with CHDs demonstrated aberrant neural activity in specific brain regions mainly related to SMN and pain processing.

### Task-based fMRI

Among the 10 studies, 5 studies reported task-based fMRI. Four studies used a block paradigm and 2-back task to evaluate the verbal working memory (VWM). The mean accuracy score for the 2-back VWM ranged between 66 ± 14% to 81.46 ± 11.41%, and the mean reaction time ranged from 981.03 ± 197.04 ms to 1110.19 ± 284.60 ms. The highest mean score obtained was 81.46 ± 11.41% (16), and the fastest mean reaction time was 981.03 ± 197.04 ms (30). Chuang et al. (26) applied an event-related executive function test, the Eriksen flanker task to assess the attention and inhibitory function and found no significant association between cardiovascular risk scores and executive function performance, based on the reaction time differences. In addition, it was noted that subjects with higher CVDs risk showed greater task-related activation in the left inferior parietal region, and it was linked to poorer task performance (26).

Meusel et al. (32) observed a suppressed DMN during the 2-back task and found that reduced DMN suppression was linked to poorer task performance. The elevation of low-density lipoprotein cholesterol (LDL-C) in those with CV led to a negative effect on the DMN function and task performance. On the other hand, a study by Chuang et al. (26) reported hyperactivity in the left parietal region in subjects with higher CVDs risk which was associated with poorer task performance. Some studies (n = 3) reported a reduction in brain activity among the CVDs subgroups. For instance, Haley et al. (16) found diminished brain activity in the right superior frontal gyrus (SFG). In addition, the authors also showed that cognitive difficulties were significantly related to weaker tb-activation, and the lower activation intensity in the right SFG was associated to poorer task performance. Another study (18) reported reduced brain activity in the insula and deduced that poor EF was related to less brain activity among patients with CVDs. This tends to be associated with slower 2-back reaction time and poorer VWM accuracy. Haley et al. (30) found that intima-media thickness (IMT) of carotid artery was associated with lower activation intensity in the right middle frontal gyrus. The 2-back task-related activation intensity in this study was positively correlated with mean accuracy but not the reaction time. Higher IMT was negatively correlated with 2-back accuracy but it showed no statistical significance. Both rs-fMRI and tb-fMRI outcomes have been elaborated in Table 4.

**Table 4:** Functional MRI outcomes and conclusive findings of included studies.

Study	fMRI	RSN	Task paradigm	Task score	Brain areas affected (ROIs)	Brain connectivity / activity	Conclusive findings
Qin et al. (31)	rs-fMRI	CD	NA	NA	Superior frontal gyrus Parahippocampal cortex Medial temporal gyrus	↑ Negative connectivity from PHC and MTG to SFG ↓ Strength of positive connectivity between PHC and MTG	Patients with CADs exhibited lower cognitive function compared to HC Alterations in the connectivity of the brain networks in CADs may mediate changes in cognitive function
Wei et al. (7)	rs-fMRI	SMN	NA	NA	Supplementary motor area Superior frontal gyrus	↓ FC patterns with the left SMA and SFG in CHDs subjects, and ↓ FC patterns with the left hemisphere for right hemisphere	Significant correlation between decreased functional lateralization of thalamic FC patterns with several subnetworks in the left hemisphere among patients with CHDs
Sun et al. (17)	rs-fMRI	DMN	NA	NA	Medial superior frontal gyrus Middle temporal gyrus Inferior occipital gyrus	↓ ReHo values in bilateral medial SFGmed and left TPOmid in CHDs ↓ fALFF values in the left and right SFGmed, left MTG and right superior temporal gyrus (TPOsup) in CHDs.	Decreased spontaneous brain activity areas in CHDs correlated with CI High CAC score correlated with poor psychological measures and cognitive dysfunction
Zhang et al. (29)	rs-fMRI	CD	NA	NA	Right precuneus gyrus Left supramarginal gyrus Left angular gyrus Left middle cingulum gyrus	↓ ALFF values in patients with CADs compared to HC	Brain regions showed ALFF differences in CADs Lower ALFF values in CADs correlated with their lower MoCA scores and reduced visual acuity
Lin et al. (25)	rs-fMRI	SMN	NA	NA	Precentral/postcentral gyrus	↓ fALFF values in the left precentral/postcentral gyrus, and ↑ fALFF values in the right inferior cerebellum	Subjects with CHDs demonstrated aberrant neural activity in specific brain regions mainly related to SMN and pain processing
Meusel et al. (32)	tb-fMRI	NA	2-back working memory task Breath-hold task	Mean 2-back task accuracy score = 66 ± 14% Mean reaction time = NR	Medial and lateral, parietal and temporal regions Right anterior cingulate and medial frontal gyrus	↓ DMN suppression was observed during the 2-back task	Reduced DMN suppression was associated with poorer task performance. Elevated LDL-C in those with cardiovascular risk had a negative impact on DMN function and task performance
Chuang et al. (26)	tb-fMRI	NA	Eriksen flanker task (executive function task) Event-related	[Control] ConSm = 645.4 ± 86.8 ms ConLg = 683.0 ± 108.7 ms [Inhibitory] ConLg = 679.8 ± 85.9 ms IncLg = 770.1 ± 112.6 ms	Bilateral middle frontal gyrus, anterior cingulate cortex, supplementary motor area, and bilateral parietal and right occipital lobe	↑ Hyperactivity in the left parietal region in subjects with higher CVDs risk	Higher CVDs risk showed increased activation in the left parietal region and it was associated with poorer task performance
Haley et al. (16)	tb-fMRI	NA	2-back working memory task Block design	Mean 2-Back task accuracy = 81.46 ± 11.41% Mean reaction time= 1110.19 ± 284.60 ms	Right superior frontal/ middle frontal gyrus, and right inferior frontal gyrus	↓ Brain activity in the right superior frontal gyrus	Cognitive difficulties were significantly related to weaker tb-activation Lower activation intensity in the right SFG was related to poorer task performance
Irani et al. (18)	tb-fMRI	NA	2-back VWM task Block design	Mean accuracy = 78 ± 9%. Mean reaction time = 1,027 ± 192 ms	Bilateral insula and supplemental motor area	↓ Brain activity in insula	Poor EF is associated with less brain activity in insula among patients with CVDs, and It tends to be associated with slower 2-back reaction time and poorer VWM accuracy
Haley et al. (30)	tb-fMRI	NA	2-back VWM task Block design	Mean accuracy = 80 ± 9% Mean reaction time = 981.03 ± 197.04 ms	Right middle frontal gyrus	↑ IMT was associated with lower activation intensity in the right middle frontal gyrus	The 2-back task-related activation intensity was positively correlated with mean accuracy but not reaction time Higher IMT was negatively correlated with 2-Back accuracy but had no statistical significance

ALFF, amplitude of low-frequency fluctuation; CAC=Coronary artery calcium; CADs, coronary artery diseases; CHDs, coronary heart diseases; CD, cannot determine; CI, cognitive impairment; CVDs, cardiovascular diseases; DMN, default mode network; fALFF=Fractional amplitude of low-frequency fluctuations; FC, functional connectivity; HC, healthy control; IMT, intimal–medial thickening; LDL-C, low-density lipoprotein cholesterol; MoCA, Montreal Cognitive Assessment; MTG, medial temporal gyrus; NA, not applicable; PHC, parahippocampal cortex; ReHO, Regional Homogeneity; ROIs, region of interest; rs-fMRI, resting-state functional magnetic resonance imaging; SFG, superior frontal gyrus; SFGmed, medial superior frontal gyrus; SMA, supplementary motor area; SMN, sensorimotor network; tb-fMRI, task-based functional magnetic resonance imaging; VBW, verbal working memory.

## Discussion

Previously, MRI studies focused on structural brain alterations in patients with CHD and revealed white matter lesions, grey matter atrophy, and changes in cerebral blood flow. However, not much is known about the functional changes in brain activity (25). Researching the potential effects of CVDs on brain function can be better explored through measurements of neural activity using fMRI. To the best of our knowledge, this is the first systematic review to investigate the cognitive impairment and brain functional changes in various CVDs subgroups identifying both task-based and resting state fMRI outcomes. CVDs influences brain health through multiple interconnected mechanisms, including reduced blood flow, inflammation, neurodegeneration, and metabolic disturbances. Understanding these pathways is crucial for developing targeted interventions to protect cognitive function in individuals with CVDs.

The use of fMRI technology in conjunction with epidemiological risk-based initiatives to identify inefficiencies in brain regions prone to the effects of CVDs may be highly beneficial in a clinical setting (18). For instance, rs-fMRI, a non-invasive, neural activity assessment method, has been widely used to measure spontaneous neural activity independent of task stimulation constraints by detecting blood oxygen level-dependent (BOLD) signals. Current research on pain mechanisms and neuropsychiatric illnesses has used ALFF and fALFF analyses extensively to demonstrate anomalies in brain activity through spontaneous neural activity (25). Data-driven techniques like ReHo and fALFF are frequently employed to measure regional spontaneous brain activity. ReHo and fALFF have been linked to both normal cognitive function in healthy persons and CI in a variety of neurological illnesses; hence, using both approaches collectively may yield more accurate results than using either method separately (17).

Based on the qualitative findings, evidence shows that subjects with CVDs tend to show more pronounced cognitive decline having reduced MMSE/MoCA scores (17, 29, 31) and poorer task performance (16, 18, 26, 30, 32); impaired brain activity at SMA associated with poor EF (18) and reduced DMN suppression linked to high LDL-C (32); lower ReHo and ALFF values (17, 25, 29); and reduced FC (7). The DMN is particularly relevant in determining the relationship between cardiovascular risk and dementia because DMN integrity and cognitive function are closely intertwined. Notably, poor task performance has been associated with failure of DMN suppression during cognitive activities, indicating ineffective allocation of attentional resources away from the DMN and towards task-related brain areas (32). The attenuated BOLD response to a VWM challenge during tb-fMRI was related to worse peripheral vascular health as measured by common carotid artery IMT in older adults with CVDs (18, 30). Reduced neuronal activity in the SFGmed was significantly correlated with worse neurocognitive ability, according to ROI-based correlation studies. The healthy subjects, however, showed no evidence of these associations. Interestingly, the relationship between the increase in CAC score and reduction in MoCA and MMSE scores was significantly mediated by the decreased regional spontaneous brain activity in the SFGmed (17). Thus, decreased neuronal activity played an important role in the patients’ poor cognitive performance, indicating that alterations in regional spontaneous brain activity, especially in relation to the SMA and SFGmed which are crucial motor-functional components of the SMN were compromised in patients with CVDs, and led to cognitive deficits. Qin et al. (31) found that alterations in the brain connectivity networks may be responsible to cause changes in patients’ cognitive performance. The absence of positive connectivity between the right SFG and PHC was observed in case of effective connectivity in CADs. Moreover, there was a significant correlation between the strength of connectivity and cognitive function (31).

Low ReHo values in the SFGmed were found to correlate with low cognitive function and CAC scores based on the mediation analysis (17). The SMN dysfunction, pain coding, and emotional and cognitive processing may be impacted by the markedly reduced fALFF values in the left precentral/postcentral gyrus in patients with CHDs. However, the presence of greater fALFF values in the right inferior cerebellum among CHDs subjects due to increased brain activity in the right cerebellum, may be attributable to the compensatory effects with regard to both motor and non-motor dysfunctions occurring as a result of the reduced brain activity in the precentral and postcentral gyri (25). Therefore, aberrant spontaneous neural activity among patients with an underlying heart condition imposes a distinct brain functional impairment pattern.

The majority of the study subjects in this review reported multiple cardiac risk factors. CVRFs like HTN, T2DM, and HCL are highly prevalent and comorbid with advancing age and can increase the risk of CI and dementia (32). Due to the presence of shared risk factors and the direct consequential effect of cardiac impairment on brain, the prevalence of CI in patients with CVDs tends to increase steadily (33). Increased activity in the inferior parietal lobe may provide a potential pathway through which cardiovascular risk factors can increase the likelihood of CI (26). A review article (10) demonstrated that in individuals without symptomatic cardiovascular, cerebrovascular, or peripheral vascular disease, the vascular risk factors (e.g., HTN, T2DM, obesity, hyperlipidaemia, and smoking) are independently linked to brain imaging changes before the clinical manifestation of disease. Accumulation of damage from increased oxidative stress and inflammation related to CVRFs in midlife projects to more severe cognitive decline in later-life and results in aberrant neural activities (32).

Mostly, the study subjects belonged to the older age group, consistent with current literature which supports that clinical CVDs and CI often coexist in the aging populations, and relates with increased risk of CI (34, 35). A community-based study in Taiwan found that healthy, middle-aged, and older adults, with moderate-to-high CVRF burden, were significantly linked to dementia and CI (verbal memory and language), indicating a strong relationship between CI and CVDs risk burden (36). The number of female subjects with CVDs was found to be almost similar to males but slightly higher. The risk of CVDs generally heightens after menopause due to decreased oestrogen levels (37), and since the majority of the participants were elderly female patients, it is more likely that they have already reached menopause.

Our findings demonstrated that race/ethnicity, education, and socioeconomic status of participants were not evenly standardised, which contributed to some inconsistencies while reporting the demographic characteristics across these studies. Even though almost all studies included individuals with intact global cognitive functioning, however, there were differences in their clinical characteristics, CVDs duration, and medication regime which may have had a potential impact on their cognitive changes and fMRI outcomes. The use of various cognitive assessment tools may influence the study findings, and was acknowledged as a study limitation. Even though mental/ psychological conditions were considered as an exclusion criterion, however, most of the studies did not mention the type of psychometric measure. Since older adults are more susceptible to developing anxiety and depression, they may require additional psycho-behavioural assessments. Furthermore, it was not clearly emphasised whether the patients were recruited from clinical or community settings, which does not give us a clear picture of the CVDs severity and therapeutic management. Another notable limitation was the cross-sectional nature of the studies which failed to identify the causal relationship between cardiovascular risk and functional brain changes. Lastly, the type of fMRI data processors used in the studies may have utilised different quantification methods to scan and analyse the functional brain images.

## Conclusion

In summary, it can be extrapolated that cardiovascular diseases tend to alter the brain network connectivity, which can lead to cognitive impairment and poorer task performance. The neural activity and fMRI patterns in the right SFG of healthy individuals typically shows robust activation during tasks requiring attention and decision-making, which is positively associated with better cognitive performance (26). In contrast, the activation in this area is often diminished in patients with CVDs, which reflects compromised cognitive processing abilities (38, 39). The difference in activation patterns between cardiac patients and healthy individuals illustrates how cardiovascular health directly impacts the brain function, particularly in regions critical for cognitive tasks. However, there may be notable exceptions and complexities that highlight the need for further research. Therefore, it is essential to continue exploring the complex relationship between cardiovascular health and brain function to better understand the mechanisms at play and to refine interventions for at-risk populations.

Cardiovascular health can directly impact brain function, particularly in regions critical for cognitive performance and goal-directed behaviour. The reduced cerebral blood flow and other vascular changes associated with CVDs tend to impair brain’s ability to engage areas like the right SFG that are necessary for complex cognitive functions (26). Overall, the collated evidence in this systematic review suggests a strong relationship between brain functional alteration and declined cognitive performance in cardiovascular diseases. The findings of this study conspicuously support that CVDs and CVRFs are associated with cognitive dysfunctionality and impaired neural activity, especially in older adults. Executive functioning, memory, and attention tend to play a vital role in medical adherence and self-care efficiency (40). Impairment in either one or more of these domains can result in a lack of adherence, follow-up, and negligence in self-care, which may eventually advance the progression of CVDs, and lead to adverse health outcomes such as repeated hospitalisation and mortality. The role of CVRFs in causing cognitive decline should be highly prioritised since positive psycho-behavioural interventions may prove to be beneficial in mitigating the modifiable risk factors and providing necessary support to the CVDs at-risk population. Cognitive screening and early identification of risk factors including vascular stiffness should be targeted at subclinical and clinical levels. This review strongly recommends future functional brain imaging studies on CVDs population with more stringent and homogenous demographic data for greater accuracy and generalizability of results.

## Data Availability

The original contributions presented in the study are included in the article/Supplementary material, further inquiries can be directed to the corresponding author.
