# Supplementary material for: Brain functional alteration and cognitive performance in cardiovascular diseases: a systematic review of fMRI studies
Source: Front Neurol. 2024 Oct 15;15:1425399. doi: 10.3389/fneur.2024.1425399 (PMC11518827; doi:10.3389/fneur.2024.1425399)
Supplement: Supplementary file 1 [file Data_Sheet_1.docx]

Table 1 Detailed search strategy and additional information

SEARCH TERMS BUILDING BLOCKS

- functional magnetic resonance imaging OR functional MRI or fMRI OR task-based fMRI or tb-fMRI OR resting state fMRI OR rs-fMRI
- cardiovascular diseases OR CVD OR heart diseases OR cardiac diseases OR coronary artery disease OR CAD OR coronary heart diseases OR CHD OR ischaemic heart disease OR IHD OR heart failure OR HF OR cardiomyopathy OR valvular disease
- cognition OR cognitive impairment OR cognitive decline OR working memory OR memory OR attention OR reaction time

MERGED SEARCH TERMS

CVD (“cardiovascular diseases” OR “heart diseases” OR “cardiac diseases” OR “coronary artery disease” OR “CAD” OR “coronary heart diseases” OR “CHD” OR “ischaemic heart disease” OR “IHD” OR “heart failure” OR “HF” OR “cardiomyopathy” OR “valvular disease”) AND fMRI (functional magnetic resonance imaging OR functional MRI OR task-based fMRI or tb-fMRI OR resting state fMRI OR rs-fMRI) AND cognition (cognitive impairment OR cognitive decline OR working memory OR memory OR attention OR reaction time)

NUMBER OF HITS

PubMed = 230 (20/04/2024)

ScienceDirect = 253 documents (22/04/2024)

WOS = 74 (25/04/2024)

ClinicalTrials.gov = 32 (26/04/2024)

TOTAL = 589

**Filters:** English and Human subjects, original research articles published until April, 2024

**Databases:** PubMed, ScienceDirect, Web of Science, ClinicalTrials.gov

Table 2 Selection Criteria for Including Studies (Eligibility criteria)

| **i.** **Population, or participants and conditions of interest** | - Adults with history of CVDs such as CAD, HF, CHD, IHD, and/or CVD risk factors (atherosclerosis, hypertension, obesity, dyslipidaemia, type II diabetes mellitus, smoking) were included. - Individuals with cerebrovascular diseases such as stroke were excluded. - Those with an underlying history of neurological disease, traumatic brain injury, psychiatric illness, dementia, substance abuse, and MRI contraindications were excluded. |
| --- | --- |
| **ii.** **Interventions or exposures** | - Functional magnetic resonance imaging (resting state fMRI and task-based fMRI) |
| **iii. Comparisons or control groups** | - Healthy adult control |
| **iv. Outcomes of interest** | **Primary outcome** (brain functional changes and neuropsychological assessment)   - RSN/ brain activity/ connectivity, cognition, working memory, attention and reaction time   **Secondary outcome**   - Demographic and clinical characteristics |
| **v. Study designs** | - **Inclusion:** original research/ prospective and retrospective/ RCT/ cross-sectional study - **Exclusion:** preprints/ conference proceedings/ systematic reviews and meta-analyses/ brief reports |

Table 3 Selection Criteria for Excluding Studies

- Articles not in English language.
- Studies conducted on children or infants.
- Studies conducted on psychiatric populations with cardiac illnesses
- Preprints/ conference abstracts/ systematic reviews/meta-analyses/ brief reports.
- Studies reporting other imaging modalities (DTI, structural MRI, cardiac MRI).
- Studies conducted on patients with stroke, cerebrovascular diseases and other medical conditions (kidney failure, congenital heart disease)
- Studies with missing information or inconsistencies in reporting of data.

Table 4 Methodological Quality Assessment of Studies

| First author (year) | S1 | S2 | Q1 | Q2 | Q3 | Q4 | Q5 | Quality |
| --- | --- | --- | --- | --- | --- | --- | --- | --- |
| Qin et al, 2023 | 1 | 1 | 1 | 1 | 1 | 0 | 1 | **** |
| Wei, 2023 | 1 | 1 | 1 | 1 | 0 | 1 | 1 | **** |
| Sun, 2022 | 1 | 1 | 1 | 1 | 1 | 1 | 1 | ********* |
| Zhang, 2022 | 1 | 1 | 1 | 1 | 1 | 1 | 1 | ********* |
| Lin, 2022 | 1 | 1 | 1 | 1 | 0 | 1 | 1 | **** |
| Meusel, 2017 | 1 | 1 | 1 | 1 | 1 | 1 | 0 | **** |
| Chuang, 2014 | 1 | 1 | 1 | 1 | 1 | 0 | 1 | **** |
| Haley, 2011 | 1 | 1 | 1 | 1 | 1 | 0 | 1 | **** |
| Irani, 2009 | 1 | 1 | 1 | 1 | 1 | 0 | 1 | **** |
| Haley, 2007 | 1 | 1 | 1 | 1 | 1 | 0 | 1 | **** |

Responses: 1 = Yes (criteria met), 0 = No (criteria not met), 0 = Can’t tell (inappropriate or unclear)

Screening questions (for all types)

S1. Are there clear research questions?

S2. Do the collected data allow to address the research questions?

Criteria

3. Quantitative non-randomized studies: Methodological quality criteria

3.1 Are the participants representative of the target population?

3.2. Are measurements appropriate regarding both the outcome and intervention (or exposure)?

3.3. Are there complete outcome data?

3.4. Are the confounders accounted for in the design and analysis?

3.5. During the study period, is the intervention administered (or exposure occurred) as intended
